# IPMiner: hidden ncRNA-protein interaction sequential pattern mining with stacked autoencoder for accurate computational prediction

**DOI:** 10.1186/s12864-016-2931-8

**Published:** 2016-08-09

**Authors:** Xiaoyong Pan, Yong-Xian Fan, Junchi Yan, Hong-Bin Shen

**Affiliations:** 1 Institute of Image Processing and Pattern Recognition, Shanghai Jiao Tong University, and Key Laboratory of System Control and Information Processing, Ministry of Education of China, Dongchuan Road, Shanghai, China; 2 Guangxi Key Laboratory of Trusted Software, Guangxi Colleges and Universities Key Laboratory of Intelligent Processing of Computer Images and Graphics, Guilin University of Electronic Technology, Guilin, China; 3 Institute of Software Engineering, East China Normal University, Shanghai, China; 4 Present Address: Department of Veterinary Clinical and Animal Sciences, University of Copenhagen, Copenhagen, Denmark

**Keywords:** ncRNA, ncRNA-protein, Deep learning, Stacked ensembing

## Abstract

**Background:**

Non-coding RNAs (ncRNAs) play crucial roles in many biological processes, such as post-transcription of gene regulation. ncRNAs mainly function through interaction with RNA binding proteins (RBPs). To understand the function of a ncRNA, a fundamental step is to identify which protein is involved into its interaction. Therefore it is promising to computationally predict RBPs, where the major challenge is that the interaction pattern or motif is difficult to be found.

**Results:**

In this study, we propose a computational method IPMiner (Interaction Pattern Miner) to predict ncRNA-protein interactions from sequences, which makes use of deep learning and further improves its performance using stacked ensembling. One of the IPMiner’s typical merits is that it is able to mine the hidden sequential interaction patterns from sequence composition features of protein and RNA sequences using stacked autoencoder, and then the learned hidden features are fed into random forest models. Finally, stacked ensembling is used to integrate different predictors to further improve the prediction performance. The experimental results indicate that IPMiner achieves superior performance on the tested lncRNA-protein interaction dataset with an accuracy of 0.891, sensitivity of 0.939, specificity of 0.831, precision of 0.945 and Matthews correlation coefficient of 0.784, respectively. We further comprehensively investigate IPMiner on other RNA-protein interaction datasets, which yields better performance than the state-of-the-art methods, and the performance has an increase of over 20 % on some tested benchmarked datasets. In addition, we further apply IPMiner for large-scale prediction of ncRNA-protein network, that achieves promising prediction performance.

**Conclusion:**

By integrating deep neural network and stacked ensembling, from simple sequence composition features, IPMiner can automatically learn high-level abstraction features, which had strong discriminant ability for RNA-protein detection. IPMiner achieved high performance on our constructed lncRNA-protein benchmark dataset and other RNA-protein datasets. IPMiner tool is available at http://www.csbio.sjtu.edu.cn/bioinf/IPMiner.

**Electronic supplementary material:**

The online version of this article (doi:10.1186/s12864-016-2931-8) contains supplementary material, which is available to authorized users.

## Background

Recently non-coding RNA (ncRNA) have received enormous attention within the field of RNA biology. ncRNAs play crucial roles in different biological processes, and their dysregulations have been associated with many human diseases [1–4]. Thousands of new ncRNAs have been discovered, whose functions have yet to be discerned. According to GENCODE v23 (released on 2015-07), around 60,000 genes have been classified for human genome, of which more than 40,000 are ncRNA genes and only 20,000 are protein coding genes, and the number of ncRNAs is increasing annually [5]. While most of the functions of ncRNAs are still unknown, therefore it is imperative to infer their functions based on their biological mechanisms. One of the known mechanisms is that ncRNA functions via interacting with proteins [6]. To get the insight into ncRNA’s functions, there is a need to identify whether this ncRNA interacts with other proteins, which can help understand the mechanism behind biological processes involving RBPs [7, 8].

There have been many promising progresses for large-scale RNA-binding protein detection, e.g. reviewed extensively in [9], such as RNAcompete [10], PAR-CLIP [11] and RNA-protein complex structure. However, these methods are still time-consuming and cost-intensive, especially in the post-genomic era. For example, experimental determination of complex structure is high-cost, and high-throughput technologies requires much time for careful hand-tuning of putatively bound sequences [12]. While there are a host of studies indicating the sequence specificities for protein-RNA interaction, they suggest that sequences carry sufficient information for predicting RNA-protein interaction [10, 12]. Hence, a reliable computational approach only from sequences is considered as a complement to identify RNA-protein interactions, such as training machine learning models to predict interactions based on accumulated experimentally verified RNA-protein pairs [13, 14]. For protein-RNA interactions prediction, some studies focus on interacting partner prediction [13–16], which predict whether an RNA can bind to a protein or not. Other studies further determine protein-RNA binding interfaces in proteins [17–19], which can identify exact binding amino acids between RNAs and proteins.

Here, we aim to computationally predict interaction partner between RNAs and proteins, which has attracted plenty of research efforts in past years [13–16, 20–22]. For instance, the catRAPID inferred lncRNA-protein association score from physiochemical properties [16, 21]. Furthermore, lncPro [15] applied Fisher linear discriminant to improve inferring association score between lncRNA and protein using features similar to catRAPID. Different from the above two approaches, some studies consider RNA-protein interaction as a classification problem. For example, Pancaldi et al. applied random forest [23] and support vector machine [24] to classify a protein and an RNA interact or not via integrating different sources of features, such as structure, localization and genomic context [20]. Simple sequence features are closely related to RNA-protein interaction [25], so RPISeq trained a random forest model only using simple 3-mer and 4-mer features from protein and RNA sequences, respectively [14]. More recently RPI-Pred combined sequences and high-order 3D structural features to identify ncRNA-protein interactions [13].

Many challenges still remain in this new area. First, in the above studies, their extracted features for proteins and RNAs were hand-crafted. As an example, in [20], the authors manually curated different sources of features, such as GO information, but only 5,166 of 13,243 positive pairs had completely available features required for model training. So more than half of positive pairs have to be discarded, which could change the real distribution behind the data. Additional file 1: Figure S1 illustrates the distribution change of significant feature Cysteine abundance from [20]. The variance of Cysteine abundance in all positives is much smaller than after discarding some positives lacking all required features, thus suggestive of low discriminant power in original data. But after discarding them, it illustrates a significant impact of Cysteine abundance on predicting RNA-protein interactions, which may lead to overoptimistic performance. On the other hand, hand-crafting discriminant features or rules for RNA-protein requires strong domain knowledge, how to select the features plays a crucial role in machine learning models.

Second, previous studies mainly extracted information from observed sequences [14–16], but they generally got lowly discriminant features because of feature noises in the observed sequences. And general machine learning models might not well handle to mine hidden associations from the noise inputs. On the other hand, for machine learning models, it is indispensable to mine refined features buried in noise inputs via multiple abstractions and refinements. Thus if we can automatically extract high-level discriminant features from some simple features based on only sequences, then the proposed method will be expected to be more robust in real-world applications.

Deep learning provides a powerful solution for this kind of problems, it consists of model architectures with multiple layers of neural network [26–28], which can extract high-level abstractions from data automatically. Meanwhile, deep learning has shown better performance than other popular machine learning methods in some research areas, such as speech recognition [29], signal recognition [26], etc. It also has been proved to be powerful in bioinformatics [12, 30, 31]. For example, deep learning has been successfully applied to predict RNA splicing patterns in and across various tissues [31]. Recently DeepBind applied deep learning to determine sequence specificities of DNA- and RNA-binding protein, which outperforms other state-of-the-art methods [12]. Similarly, DeepSEA learned regulatory sequence code from chromatin-profiling sequences using deep learning, which further prioritized functional variants [30]. In summary, deep learning has the following advantages over other sequence-based methods: 1) It can automatically learn specific sequence motifs for RNA-protein [12], and those sequence motifs have been found to directly mediate sequence-specific associations between RNAs and proteins [32, 33]. 2) It is able to reduce the impact of noises in the original data and learn real hidden high-level features [29]. Furthermore, some deep learning-based methods even artificially introduce noises to reduce over-fitting, which can enhances model generalization and robustness [34].

In this study, we propose a fully sequence-based method, IPMiner, to predict ncRNA-protein interaction using deep learning. First, it extracts raw sequence composition features from RNA and protein sequences, then applies stacked autoencoder to extract hidden high-level features [35], which are then fed into random forest to predict RNA-protein interactions. Furthermore, stacked ensembing is used to integrate different predictors to improve the model performance. Our contributions are summarized as follows: (1) The newly designed network architectures can automatically extract abstraction features from sequence composition features of proteins and RNAs, and is able to learn sequence specificities for proteins and RNAs, respectively. (2) We applied deep learning to better fuse the learned high-level features from raw input features of proteins and RNAs, instead of directly concatenating them to be fed into classifiers. (3) We introduced another logistic regression classifier layer based on the intuition behind deep learning to integrate the predictions from different methods, which improves the IPMiner’s performance.

The experiments on our constructed lncRNA-protein benchmark dataset from Protein Data Bank (PDB) [36] demonstrate that IPMiner achieves high performance. Besides, we also test our method IPMiner on previous published datasets, such as RPI1807 [13], RPI369 and RPI2241 [14], RPI13254 [20, 37] and NPInter2.0 database [38], and IPMiner yields better performance in all datasets than other sequence-based methods RPISeq-RF [14] and lncPro [15].

## Results

**Fig. 1:**
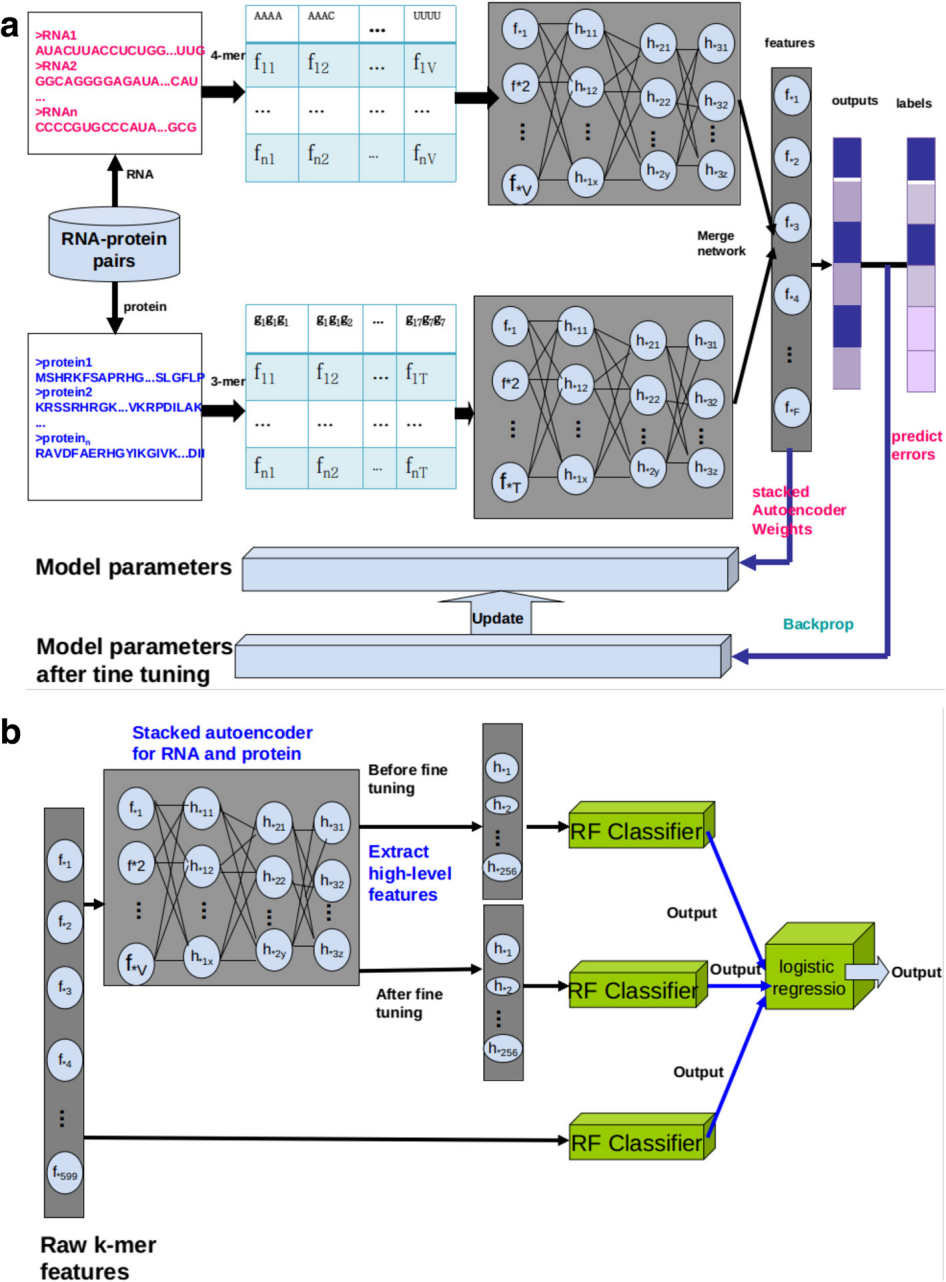
The flowchart of proposed IPMiner. It proceeded in two main steps. **a** Train stacked autoencoder models for RNA and protein, respectively, and fine tuning for it using label information from RNA-protein pairs. **b** Apply stacked ensembling to integrate SDA-RF, SDA-TF-RF and RPISeq-RF, which used high-level features before fine tuning, high-level features after fine tuning and raw k-mer frequency features, respectively. The network architectures were 256-128-64 with 256, 128, and 64 neurons in 3 hidden layers for stacked autoencoder

In this study, we proposed IPMiner (Fig. 1), stacked ensembling of SDA-RF, SDA-FT-RF and RPISeq-RF, for predicting lncRNA-protein interactions, where the RF stands for random forest, the SDA stands for stacked denoising autoencoder, and the SDA-FT stands for stacked denoising autoencoder with fine tuning. Meanwhile we also tested the performance of SDA-RF, SDA-FT-RF, RPISeq-RF and lncPro on different datasets, including structure-based RPI488, RPI1807, RPI2241 and RPI369, and non-structure-based NPInter2.0, RPI367 [39], RPIntDB (http://pridb.gdcb.iastate.edu/RPISeq//download.php) [40] and RPI13254. Considering unavailability of RPI-Pred and catRAPID standalone, here we only compared IPMiner with RPISeq-RF and lncPro [15]. lncPro only provides a prediction source code based on the trained model on their dataset, which overlaps with our constructed data RPI488 collected in this study. In addition, we focused on classification performance. To make it work for classification and be comparable with IPMiner, we adapted lncPro’s source code. We only used the extracted features for RNAs and proteins after Fourier series transformation from lncPro, then feed them into random forest to evaluate the performance.

### Comparison between different layer architectures for IPMiner

To investigate the impact of different network architectures on IPMiner’s performance, we also designed two different network architectures with fully connected layer:

1) Sep-256-128-64: It has two separate (Sep) stacked networks. One is for proteins, the other one is for RNAs, their inputs are protein sequence features and RNA sequence features, respectively. And the last hidden layer is the concatenation of the two sub-networks. The hidden layers for two stacked sub-networks are both 256-128-64. Here 256-128-64 means that the number of neurons for 3 hidden layers in stacked autoencoder are 256, 128, and 64, respectively.

2) Con-256-128-128, The raw input is concatenation (Con) of protein and RNA sequence features, which connects to one stacked networks. The hidden layers for stacked autoencoder is 256-128-128, whose 3 hidden layers have 256, 128, and 64 neurons, respectively.

From Table 1, we can see that Sep-256-128-64 yielded over 2 % higher accuracy than Con-256-128-128, which indicated that when learning sequence specificities for RNAs and proteins, neurons from RNA and protein should not connect to the same neurons in successive layers. Otherwise the information hidden in proteins and RNAs will corrupt with each other. The results demonstrated that RNA and protein k-mer features should have no interaction with each other, and stacked autoencoder can automatically learn sequence specificities inside proteins and RNAs, respectively.

**Table 1:** Performance comparison between different layer architectures on RPI488

Architecture	Method	Accuracy	Sensitivity	Specificity	Precision	MCC	AUC
Sep-256-128-64	IPMiner	**0.891**	**0.939**	0.831	**0.945**	**0.784**	**0.914**
	SDA-RF	0.880	0.922	0.827	0.928	0.762	0.904
	SDA-FT-RF	0.881	0.916	0.831	0.926	0.762	0.909
Con-256-128-128	IPMiner	0.872	0.893	**0.843**	0.894	0.743	0.903
	SDA-RF	0.884	0.924	0.831	0.934	0.770	0.911
	SDA-FT-RF	0.864	0.885	0.836	0.887	0.727	0.898
Raw input	RPISeq-RF	0.880	0.926	0.822	0.932	0.762	0.903
Raw input	lncPro	0.870	0.900	0.827	0.910	0.740	0.901

Raw input is concatenation of 3-mer frequency features of protein and 4-mer frequency features of RNA

The boldface indicates this measure performance is the best among the compared methods for individual dataset

Different network architectures were trained on our constructed dataset using the different number of neurons in the hidden layers. The results shown in Additional file 2: Table S2 indicated that 256-128-64 achieved better performance.

### IPMiner achieved high performance for predicting lncRNA-protein interactions

**Fig. 2:**
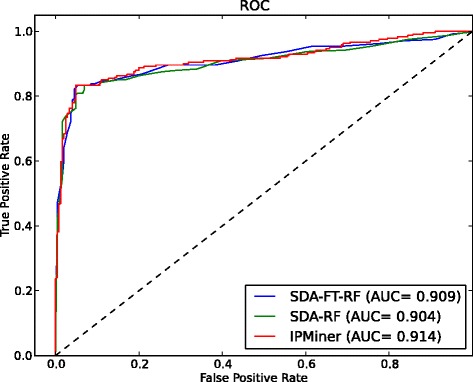
Performance on RPI488. Performance comparison between IPMiner, SDA-FT-RF and SDA-RF on lcnRNA-protein dataset RPI488

We first tested IPMiner on our own constructed lncRNA-protien interaction dataset RPI488. The ROC curve shown in the Fig. 2 showed the comparison between the performance of IPMiner, SDA-FT-RF and SDA-RF. All the three methods achieved high performance with an AUC greater than 0.90, IPMiner performed a little better than the other methods. From Table 1, it yielded an accuracy of 0.891, sensitivity of 0.939, specificity of 0.831, precision of 0.945 and MCC of 0.784, which was better than PISeq-RF with an accuracy of 0.880, sensitivity of 0.926, specificity of 0.822, precision of 0.932 and MCC of 0.762, respectively. On the other hand, for individual predictors, SDA-RF, SDA-FT-RF and RPISeq-RF perform differently in different measures. SDA-FT-RF obtained the best accuracy and specificity, RPISeq-RF got the best sensitivity and precision. This implied that they have lower correlation on predicted interactions, which is very promising for combining them together. The reason is that the more diversity the base predictors have, the better the accuracy of the ensemble predictor achieves [41], which was proved by IPMiner’s performance.

We further compared IPMiner with lncPro on RPI488, lncPro yielded an accuracy of 0.870, sensitivity of 0.900, specificity of 0.827, precision of 0.910 and MCC of 0.740, which was a little worse than IPMiner, and a little better than PRISeq-RF (Table 1). However, compared to IPMiner and PRISeq-RF, lncPro has some disadvantages: 1) It cannot predict for protein sequence shorter than 30, which is required by protein structure prediction tool predator [42]. 2) It took long time to predict RNA structure, especially for long RNA sequence, using RNAsubopt [43]. In addition, the RNA sequence must be shorter than 4095, otherwise the RNAsubopt software will only process the first 4095 nucleotides. The above is also the reason that we do not include lncPro in our ensemble predictor IPMiner. One of IPMiner’s merit is that it directly extracts low-level features from sequences, which does not depend on other prediction tools and is applicable to any protein and RNA pairs.

We also tested IPMiner on two lncRNA-protein datasets (RPI419 and RPI325) with lower sequence similarity, which both have RNA sequence similarity cut-off 80 %, but different protein sequence similarity cut-off (50 % and 30 %, respectively). As indicated in Additional file 3: Figure S2, IPMiner achieved the AUC of 0.891 and 0.881 on RPI419 and RPI325, respectively, which was a little worse than RPI488 with the AUC of 0.914, indicating sequence similarity have limited impact on IPMiner and does not lead to an inflated estimate of the predictive performance.

### Comparison between different ensembling strategy

**Fig. 3:**
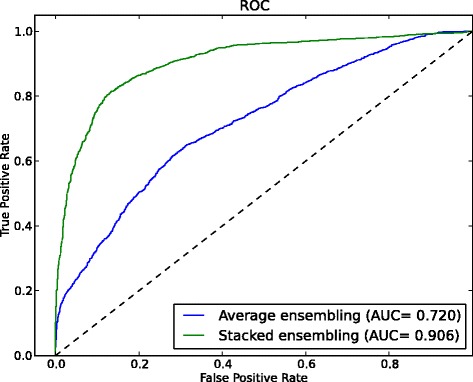
Ensembing strategy. Performance comparison between stacked ensembling and average ensembling on dataset RPI2241

In IPMiner, we applied stacked ensembling strategy to integrate different predictors, here we compared it with general averaging ensembling (averaging the predicted probability of SDA-RF, SDA-FT-RF and RPISeq-RF). As shown in Fig. 3, stacked ensembling achieved the AUC of 0.906 on RPI2241 dataset, it increased by 26 % over averaging ensembling with the AUC of 0.720. When using logistic regression to integrate the outputs from 3 predictors, it got weights 10.56, -3.77 and 1.77 for SDA-FT-RF, SDA-RF and RPISeq-RF, respectively. The contribution of SDA-FT-RF was approximately 6 and 3 times more than RPISeq-RF and SDA-RF, respectively, which implied that different predictors contributed to final combined result differently. On RPI369 dataset, the 3 predictors had smaller difference than on RPI2241 (Table 2), implying the predictors on RPI369 have higher correlation than on RPI2241. Stacked ensembing (AUC of 0.773) improved the AUC with higher margin than averaging ensembing (AUC of 0.725) on RPI369, shown in Additional file 4: Figure S3. But compared to the improvement on RPI2241, it is relatively smaller. The results indicated that stacked ensembling is very promising for improving the performance from different predictors, especially for those with lower correlation.

**Table 2:** Performance comparison on structure-based RPI369, RPI2241 and RPI1807

Dataset	Method	Accuracy	Sensitivity	Specificity	Precision	MCC	AUC
RPI2241	IPMiner	**0.824**	0.833	**0.812**	0.836	**0.650**	**0.906**
	SDA-RF	0.648	0.653	0.630	0.665	0.296	0.687
	SDA-FT-RF	0.783	**0.890**	0.645	**0.920**	0.592	0.898
	RPISeq-RF	0.646	0.652	0.630	0.663	0.293	0.690
	lncPro	0.654	0.659	0.640	0.669	0.310	0.722
RPI369	IPMiner	**0.752**	**0.735**	**0.791**	**0.713**	**0.507**	**0.773**
	SDA-RF	0.707	0.699	0.727	0.689	0.416	0.754
	SDA-FT-RF	0.693	0.664	0.784	0.602	0.396	0.728
	RPISeq-RF	0.704	0.705	0.702	0.707	0.409	0.767
	lncPro	0.704	0.708	0.696	0.713	0.409	0.740
RPI1807	IPMiner	**0.986**	**0.982**	0.993	**0.978**	**0.972**	**0.998**
	SDA-RF	0.972	0.970	0.981	0.962	0.944	0.995
	SDA-FT-RF	0.972	0.955	**0.997**	0.940	0.944	0.995
	RPISeq-RF	0.973	0.968	0.984	0.960	0.946	0.996
	lncPro	0.969	0.965	0.981	0.955	0.938	0.994

The positive pairs are all from original papers. The negative pairs for RPI1807 is from original paper

The boldface indicates this measure performance is the best among the compared methods for individual dataset

### Comparing IPMiner with other methods

To test the reliability and robustness of IPMiner, we also compared it with other sequence-based methods RPISeq and lncPro on other published ncRNA-protein and RNA-protein datasets. In study [14], the authors proposed RPISeq-RF and RPISeq-SVM for predicting RNA-protein interaction, and RPISeq-RF performed better than RPISeq-SVM on both RPI369 and RPI2241. Accordingly here we only compared IPMiner with RPISeq-RF.

As shown in Table 2, for individual predictors on RPI2241, SDA-FT-RF achieved the best performance with an accuracy of 0.783, sensitivity of 0.890, specificity of 0.645, precision of 0.920 and MCC of 0.592, which indicated that fine tuning can improve extracting complex abstraction features, and it increased the accuracy with 20 % over baseline RPISeq-RF (0.646). On RPI369, SDA-RF obtained the best performance with a little advantage over RPISeq-RF and SDA-FT-RF. And on RPI1807, RPISeq-RF yielded a little better performance than SDA-RF and SDA-FT-RF. In summary, SDA-FT-RF achieved similar performance with slightly worse accuracy on some datasets, but it can improve the performance on certain datasets a lot.

On the other hand, the above results indicated that individual predictors can perform differently on different datasets, and no single predictors can surpass others in all datasets. So IPMiner applied stacked ensembing to integrate different predictors. As indicated in Table 2, IPMiner was superior to all individual methods on all datasets, and improved a lot over individual predictors in some datasets. On RPI2241, IPMiner yielded an accuracy of 0.824, which increased about 5 %, 27 %, 28 % over single predictor SDA-FT-RF (0.783), SDA-RF (0.648) and RPISeq-RF(0.646), respectively. IPMiner achieved a prediction accuracy of 0.752 with an increase of about 7 % over all single predictors on RPI369, and an accuracy of 0.986 with an increase of 1 % over all single predictors on RPI1807. The results showed that stacked ensembing can improve the prediction performance on all datasets, demonstrating the effectiveness for integrating different predictors.

In addition, we also compared IPMiner with lncPro on these 3 datasets as shown in Table 2. The results indicated that lncPro performed worse than IPMiner among all 3 datasets. Especially on RPI2241, lncPro yielded a MCC 0.310, much worse than MCC 0.650 of IPMiner. Meanwhile lncPro performed a little better than PRISeq on RPI2241 and RPI369, but a little worse on RPI1807, it also indicated that individual predictors performed differently on different datasets.

For RPI369 and RPI2241, it is worth mentioning that RPISeq-RF performed worse on our constructed dataset than [14] on their own dataset, which had the same positive pairs but different selected negative pairs, indicating different negative datasets had an important impact on model performance. However, on RPI1807, using their provided positive and negative pairs, IPMiner and RPISeq-RF yielded much better performance. RPISeq-RF achieved the accuracy of 0.973, which was much better than RPI-Pred with the accuracy of 0.83 using sequence and predicted structure, even better than the accuracy of 0.93 using sequence and experimentally determined structure [13].

### IPMiner’s performance on large-scale non-structure-based experimental data

To evaluate our proposed method on other different sources of RNA-protein interaction datasets, we downloaded another two larger non-structure-based experimental datasets: One is NPInter2.0, consisting of 10412 experimentally verified ncRNA-protein pairs from 6 model organisms [38]. The other one was RPI13254 studied by [20], which is based on published interactions from [37]. It covered 13254 positive pairs and 5172 negative pairs. Here we created a balanced training dataset via randomly down-sampling 5172 positive pairs from positive dataset for 5-fold cross-validation.

As shown in Table 3, for NPInter2.0 dataset, RPISeq-RF yielded a better accuracy than any other individual predictors, which was better than SDA-RF (0.937), SDA-FT-RF (0.934) and lncPro(0.928). But on RPI13254, SDA-FT-RF got the best performance (0.813) with huge improvement over SDA-RF (0.699),RPISeq-RF (0.739) and lncPro(0.712), which also implied that there was no single predictors defeating others on non-structure-based datasets, which was similar to structure-based datasets.

**Table 3:** Performance comparison on non-structure-based NPInter2.0 and RPI13254

Dataset	Method	Accuracy	Sensitivity	Specificity	Precision	MCC	AUC
NPInter2.0	IPMiner	**0.952**	0.946	**0.959**	0.945	**0.904**	**0.995**
	SDA-RF	0.937	0.940	0.935	0.941	0.876	0.975
	SDA-FT-RF	0.934	**0.953**	0.912	**0.955**	0.868	0.990
	RPISeq-RF	0.944	0.940	0.949	0.940	0.889	0.978
	lncPro	0.928	0.919	0.938	0.917	0.856	0.971
RPI13254	IPMiner	**0.945**	**0.905**	0.995	**0.895**	**0.896**	**0.985**
	SDA-RF	0.699	0.717	0.658	0.741	0.400	0.761
	SDA-FT-RF	0.813	0.728	**0.998**	0.626	0.675	0.901
	RPISeq-RF	0.739	0.766	0.688	0.790	0.480	0.817
	lncPro	0.712	0.716	0.701	0.723	0.424	0.792

For RPI13254, it has 13524 positive pairs and 5172 negative pairs. Here we randomly sub-sampling positive pairs from original paper to create balanced dataset, so it actually consists of 5172 negative pairs and 5172 positive pairs

The boldface indicates this measure performance is the best among the compared methods for individual dataset

In addition, IPMiner was still superior to all single predictors on both datasets, it achieved the high accuracy of 0.952, 0.945 on NPInter2.0, RPI13254 respectively. Especially for RPI13254 dataset, It was an increase of about 28 % over RPISeq-RF (0.739), and stacked ensembling increased the accuracy from 0.813 of best individual predictor to 0.945 at large margin. Meanwhile it also had a very huge improvement compared to the previous reported accuracy of 78 % using RF classifiers with different sources of features [20]. The above results convinced that IPMiner can also be applied for non-structure-based experimental data.

### Predicting ncRNA-protein interactions using IPMiner

To verify IPMiner’s ability of predicting RNA-protein interaction, we further investigated the performance of our trained model from RPI488 on NPInter2.0, RPI367 and RPIntDB dataset. There is no overlapped interaction pairs between RPI488 and the 3 datasets. For NPInter2.0, IPMiner yielded the promising predictions of interactions, it correctly predicted 96.7 % of total interactions, which is better than 90 % of RPI-Pred [13]. As shown in Table 4, IPMiner predicted 97.6 %, 61.1 %, 96.2 %, 96.7 %, 94.5 %, 87.1 % for Homo sapiens, Caenorhabditis elegans, Mus musculus, Drosophila melanogaster, Saccharomyces cerevisiae and Escherichia coli, respectively. It also yielded a similar performance on RPI367 for different species and predicted 90.1 % of total interactions. The results on both datasets indicated that IPMiner is very promising for predicting ncRNA-protein interactions.

**Table 4:** The predicted performance of trained model from RPI488 on NPInter2.0, RPI367 and RPIntDB dataset

Dataset	Organism	Total # of	Predicted # of
		ncRNA-protein	ncRNA-protein
NPInter2.0	Homo sapiens	6,975	6,809 (97.6 %)
	Caenorhabditis elegans	36	22 (61.1 %)
	Mus musculus	2,198	2,115 (96.2 %)
	Drosophila melanogaster	91	88 (96.7 %)
	Saccharomyces cerevisiae	910	860 (94.5 %)
	Escherichia coli	202	176 (87.1 %)
	Total	10,412	10,070 (96.7 %)
RPI367	Homo sapiens	148	132 (89.2 %)
	Caenorhabditis elegans	2	2 (100.0 %)
	Mus musculus	46	34 (73.9 %)
	Drosophila melanogaster	26	24 (92.3 %)
	Saccharomyces cerevisiae	119	117 (98.3 %)
	Escherichia coli	25	21 (84.0 %)
	Total	366	330 (90.1 %)
RPIntDB	Total	44,586	38,522 (86.4 %)

For NPInter2.0, RPI-Pred can predict 90 % of total interactions [13]. If proteins and RNAs in a pair are obsolete, then this pair will be removed. For example, in RPI367, protein O16646 is obsolete in UniProtKB, and ncRNA u1136 interacts with O16646, this pair was removed in RPI367. In RPIntDB, there is no organism information for some interaction pairs, so we only report the total prediction accuracy

Furthermore, IPMiner correctly predicted 86.4 % of all interactions on the largest dataset RPIntDB with 44,586 interactions [14, 40], which was integrated from different sources of RNA-protein interactions, such as RNA-protein complexes, literature mining and NPinter2.0. And PRISeq-RF was able to correctly predict 81.6 % of them, which was lower than IPMiner. The results on this large-scale dataset also indicated the power of IPMiner.

In addition, we ran CD-HIT tool to reduce sequence identity between the testing datasets and RPI488. Take NPInter2.0 for example, we removed similar sequences using CD-HIT against RPI488, so that there were no sequences with sequence similarity greater than 80 % for protein and RNA sequences between NPInter2.0 and RPI488. Then we removed those interaction pairs whose protein or RNA has sequence identity greater than 80 % with RPI488. Finally, the number of interaction pairs for NPInter2.0 are reduced from 10,412 to 10,350. We tested the non-redundant 10,350 pairs using our trained model on RPI488, it yielded an accuracy of 95.7 %, which was a little lower than 96.7 % on original NPInter2.0. The results shown in Additional file 2: Table S3 indicated that there is only minor performance difference after removing minor part of redundant interaction pairs. The same processes were also done for RPI367 and RPIntDB (Additional file 2: Table S3).

### Constructing ncRNA-protein network using predicted scores from IPMiner

**Fig. 4:**
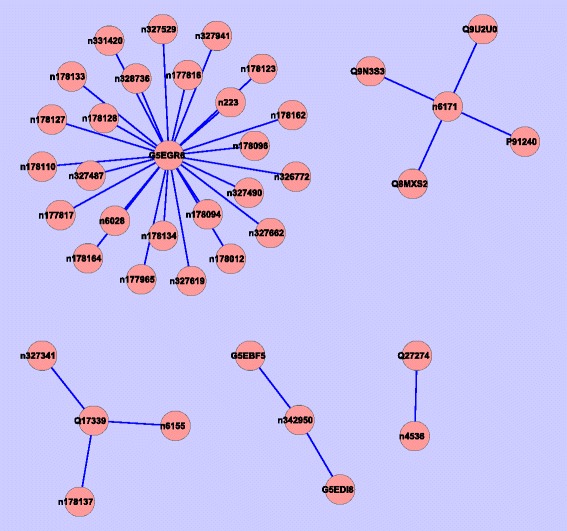
Interaction network. Clusters of MCL clustering from ncRNA network constructed from predicted ncRNA-protein pairs using IPMiner for Caenorhabditis elegans

We further applied our trained model from RPI488 on NPInter2.0 dataset to construct network for ncRNAs and proteins, which can be used to infer the functions of ncRNAs. For constructing network, we represented ncRNA-protein pairs as a weighted network, where the edge weight between ncRNA and protein was predicted probability from IPMiner, then we used Markov cluster (MCL) algorithm [44] to do clustering on the constructed ncRNA-protien network. For Caenorhabditis elegans in NPInter2.0, IPMiner correctly identified 22 of 36 interactions, then we constructed ncRNA-protein network based on the similarity score from IPMiner. The corresponding clusters after MCL clustering on the constructed network were shown in Fig. 4, we found a hub protein G5EGR6 interacting 26 ncRNAs and a hub ncRNA n6171 (snRNA Z81105) interacting with 4 proteins [45], which was experimentally verified using CLIP-Seq data [46].

## Discussion

In this study, we presented a computational method based on deep learning and stacked ensembing to predict ncRNA-protein interactions. It achieved an accuracy of 0.891, sensitivity of 0.939, specificity of 0.831, precision of 0.945 and MCC of 0.784 on our constructed lncRNA-protein dataset, respectively. Comprehensive experimental results on other previous published datasets also were indicative of the effectiveness of IPMiner. On some datasets, it improved the model performance with an increase of roughly 20 % over other existing sequence-based methods. The results also indicated that stacked autoencoder can extract the discriminant high-level features, which is very crucial for building machine learning models. Where high-level features are the features automatically learned from multiple layers of neural network.

IPMiner demonstrated good performance both on ncRNA-protein and RNA-protein prediction, which is better than the state-of-the-art methods. It is mainly due to the following reasons:

1. The k-mer frequency itself indicated sequence-binding preference of RBPs bound by ARE-binding proteins [25], where over-represented k-mers in sequences can be enrich motifs for RNA-protein binding [47]. The more frequent this k-mer sequence exists in a subset of sequences, the higher probability it is a binding motif.
2. Deep learning is able to model complicate statistical characteristics in data [12, 30]. k-mer feature is similar to vocabulary word in document, and deep learning can extract the abstraction features like topics in articles from bag of words [48]. So stacked autoencoder can automatically learn hidden relationship between k-mer sequence motif, it will extract the most informative high-level features from its compressed representation, which avoids curse of dimensionality via eliminating hidden irrelevant variabilities, especially for the high-dimensional raw k-mer input features. As shown in DeepBind [12], deep learning can automatically capture the specificities of binding motifs using convolutional filters, DeepSEA [30] learned regulatory motifs from sequences, which both play a crucial role in RNA-protein binding.
3. Different predictors have different performances on different datasets, no single methods can surpass others in all datasets. Different from manually designed average voting or majority voting, stacked ensembing can better integrate the strengths of individual predictors, which is implemented in the form of adding another classifier layer to automatically figure out how to fuse the outputs from individual predictors.

RNA-protein interaction network can offer deep insights into RNA cellular mechanisms [9, 49]. For example, LPN [50] constructed a lncRNA-protein network using experimentally verified interactions, and found the potential co-regulation and functional link among lncRNAs, which were also associated with diseases or cancer pathways. However, currently only a minor part of lncRNA-protein interactions in nature are experimentally verified. To obtain a global view of interaction network, more and more interaction data should be integrated, including experimental detection and computational prediction. We expect IPMiner to be integrated to construct large-scale network to discover the functions of lncRNAs and other biological mechanisms.

Currently our method is still trained on small dataset because of the difficulties to collect large-scale experimentally verified lncRNA-protein pairs from complex structures in PDB. On the other hand, deep learning is expected more powerful on big dataset, then it will automatically learn more representative features [51]. So we need construct larger training dataset to cover all possible situations. For constructing training data, we can collect positive dataset from structure complexes and other experimental methods. Nevertheless, it is very hard to verify negative pairs in nature, accordingly in general they are generated by pairing RNA and protein randomly to get a balanced dataset with the same number of negative pairs. The rational behind constructing the balanced training dataset is that machine learning model has preference to dominant class when the data is unbalanced [18]. On the other hand, the trained model could still be biased trained on this dataset, it is because the negative dataset only cover small part of possible negative pairs in nature, and some of them could be still unverified interacting pairs. Negative control data set had the impact on the constructed prediction models, as shown in RPI2241 and RPI369. RPISeq-RF performs worse on training dataset with only different selected random pairs, which indicates the negative samples are important for the performance. In future work, we will figure out a suitable approach for a better learning from the negative sample distributions. Another strategy to cope with sample unbalance is to train models on positive samples without negative samples. For example, PRIPU trains a biased SVM on only positive and unlabelled examples [52].

Although IPMiner can achieve much better performance, there are still some limits similar to other machine learning-based approaches. It is a black box learning algorithm, and does not provide biological insights into ncRNA-protein interactions. IPMiner tries to automatically capture high-level features using deep neural network, and those learned high-level features have strong discriminate power but are still not well explained from biological perspectives. In future work, we expect to design better network architectures to learn high-level features with biological insights, such as introducing convolutional neural network to capture regulatory motifs [12].

## Conclusion

In this study, we presented a computational method IPMiner to mine the hidden interaction patterns between ncRNAs and proteins, which is based on stacked autoencoder, and further improves the performance by stacked ensembling. From simple sequence composition features, IPMiner can automatically learn high-level abstraction features, which has strong discriminant ability for RNA-protein detection. IPMiner achieved high performance on our constructed benchmark lncRNA-protein dataset. Meanwhile, comprehensive experimental results on other RNA-protein datasets also indicated that it can yield better performance than other state-of-the-art methods.

## Methods

### Data source

RNA can be catalogued into mRNA and non-coding RNA (ncRNA), where ncRNA includes small ncRNA, such as miRNA, snoRNA, and long ncRNA (lncRNA), whose size is longer than 200nt. Different RNAs have different biological functions, but their binding motifs may be similar. To verify the robustness of IPMiner, we validate it on different RNA-protein interactions datasets, including mRNA-protein and lncRNA-protein datasets. Currently there are relatively fewer study about lncRNA than mRNA, and the mechanism and functions of lncRNAs remain largely unknown, but lncRNAs are increasingly being studied.

Firstly we downloaded 18 ncRNA-protein complexes according to [15] from the Protein Data Bank (PDB) database [36]. 10 of the complexes are X-ray structures, and 8 of them is from Electron microscope, the details are listed in Additional file 2: Table S1. We used the full sequences of proteins and RNAs found in PDB structure. They are extracted from the “sequence field" of PDB file instead of the fragments having coordinates, which consists of the full sequences and are the same as the sequences in UniProt and GeneBank. The reason why we used full sequences instead of fragment sequences is that IPMiner is a sequence-based ab-initio predictor and does not need the 3D coordinates as the feature inputs. Then 726 lncRNA-protein pairs were collected from these complexes. In order to determine whether a pair is interactive or non-interactive, we used the *least atom distance* as the criterion [53]: if there exists an atom of lncRNA and an atom of protein such that the distance between these two atoms is less than the distance cutoff 5Å [53], the pair (lncRNA and protein) is considered to be interactive. Otherwise, the pair is non-interactive. After each pair in the dataset was checked, the redundant dataset including 383 interactive pairs and 343 non-interactive pairs was obtained. In order to reduce the bias of sequence homology, the redundant sequences with sequence similarity greater than 90 % (used in [15]) for both protein and lncRNA sequences were excluded by using CD-HIT tool [54]. After redundancy removal, a dataset containing 488 protein-lncRNA pairs, including 243 interactive pairs and 245 non-interactive pairs, was obtained. This dataset was called the non-redundant RPI488 dataset. Here we only got 243 lncRNA-protein interactions, which is smaller than other RNA-protein datasets, it is because that there are much fewer lncRNA-protein complexes in PDB.

Besides, to see the impact of sequence similarity on IPMiner, we also constructed another two datasets with lower sequence similarity. For the first dataset called RPI325, the sequence similarity for protein is smaller than 30 % and for RNA is smaller than 80 % (the smallest cut-off value 80 % can be configured for RNA sequence in CD-HIT tool), and we obtained 325 lncRNA-protein pairs consisting of 153 interactive pairs and 172 non-interactive pairs. In the second dataset called RPI419, the sequence similarity for protein is smaller than 50 % and for RNA is smaller than 80 % like RPI325, we obtained 419 lncRNA-protein pairs consisting of 203 interactive pairs and 216 non-interactive pairs.

To test the robustness of IPMiner, we also collected other RNA-protein datasets from the previous studies, such as RPI1807 [13], RPI369 and RPI2241 [14], RPI13254 [20, 37] and NPInter2.0 database [38], whose details are shown in Table 5. For RPI369, RPI2241, RPI1807 and RPI488, they were all extracted based on structure-based experimental complexes. On the other hand, rather than from structure-based experimental complexes, NPInter2.0 and RPI13254 are obtained from other physical association between ncRNAs and proteins [37, 38]. For constructing non-interaction pairs, the same number of negative pairs were generated by randomly pairing proteins with RNAs and further removing the existing positive pairs [14].

**Table 5:** The number of RNA-protein interaction pairs in collected datasets

Dataset	# of	# of RNAs	# of	Reference
	interaction pairs		proteins	
RPI1807	1807	1078	1807	[13]
RPI369	369	332	338	[14]
RPI2241	2241	842	2043	[14]
NPInter2.0	10412	4636	449	[38]
RPI13254	13254	4500	42	[37]
RPI488	243	25	247	This study

RPI488 is lncRNA-protein interactions based on structure complexes, PI369, RPI2241, RPI1807 are RNA-protein interactions. NPInter2.0 and RPI13254 are ncRNA-protein interactions from non-structure-based source

### Conjoint triad (3-mer frequency) feature for protein and 4-mer frequency feature for RNA

To obtain raw features for stacked autoencoder, we extracted simple sequence component composition features both for RNAs and proteins. Conjoint triad (3-mer) of protein is composed by 3 amino acids [14]. Firstly the 20 amino acids were reduced into 7 groups based on their dipole moments and side chain volume: (Ala, Gly, Val), (Ile, Leu, Phe, Pro), (Tyr, Met, Thr, Ser), (His, Asn, Gln, Tpr), (Arg, Lys), (Asp, Glu) and (Cys) [13, 55]. Then protein sequence is reduced to 7-letter alphabet, the frequency of conjoint triad features based on 7 reduced letters were extracted for each protein sequence, we got 7 ×7×7 = 343 dimensional features. Similarly, we extracted 4-mer frequency for RNA sequence (A,C,G,U), and we got 4 ×4×4×4 = 256 dimensional features [14], each feature value is the normalized frequency of 4-mer nucleotides in RNA sequences, which is AAAA, AAAC…TTTT.

### IPMiner overview

In this study, we proposed IPMiner to predict ncRNA-protein interactions, it proceeds in the following phases: 1) Extract conjoint triad (3-mer) from protein sequences and 4-mer frequency from RNA sequences; 2) Apply stacked autoencoder to extract high-level features, called SDA, from the extracted sequence features of RNAs and proteins, respectively. So two sub-networks for protein and RNA are generated; 3) Add another softmax layer to merge the two sub-networks of RNA and protein, and then use label information of training data for fine tuning the above stacked autoencoder, update the weights of networks and extracted features from updated stacked autoencoder, the new feature is called SDA-FT; 4) Feed the extracted raw features, SDA and SDA-FT features to random forest classifier, respectively, and the 3 classifiers are named as RPISeq-RF [14], SDA-RF and SDA-TF-RF, respectively; 5) Use stacked ensembling to integrate the outputs from the above 3 classifiers, which trains a logistic regression model on the outputs from them.

The flowchart of proposed IPMiner is shown in Fig. 1.

### Stacked autoencoder

Deep learning [27, 28] is widely applied in different areas with record-breaking performance [12, 28]. Autoencoder network can be used as a building block for deep network with multiple layers.

Assume we have an input data **x** with d-dimension, autoencoder network first map the **x** into **y**.

1 $$ \mathbf{y} = f(\mathbf{Wx} + \mathbf{b})  $$

where *f* is a non-linear function. After this mapping is done, the embedding **y** is mapped back to reconstruction **z** of the same shape as **x**, which is performed as follows:

2 $$ \mathbf{z} = g\left(\mathbf{W^{T}y} + \mathbf{b}^{\prime}\right)  $$

where *g* is another non-linear function, and the weights of two mappings have the constraint *W*^*T*^=**W**

The reconstruction error can be measured using squared error between **x** and **z**, which can be optimized using stochastic gradient descent (SGD) [56].

Stacked autoencoder is a deep network formed from stacking multiple autoencoders [35]. It can automatically learn high-level features that form a good representation for data from raw simple features. In general, it is organized in sequential layer-by-layer structure with multiple layers of neural networks, in which each layer contains designed number of neurons, and the outputs of each layer is connected to the inputs of the successive layer.

When learning the parameters of stacked autoencoder, it optimizes objective function using greedy layer-wise learning, which learns each layer individually while freezing parameters of other layers. To produce better performance, after this unsupervised learning, fine-tuning based on back-propagation is used to tune the parameters of all layers. It is supervised learning phase, which can improve stacked autoencoder a lot.

The layer types used in our model are fully connected layer and dropout layer [57]. For dropout layer, it randomly set some unit activations with certain probability to zero, which can avoid over-fitting for model training. For fine tuning, we add a last softmax layer with sigmoid function as activations for the outputs from merged sub-networks of protein and RNA as the last hidden layer, which is trained using label information to update weights and biases parameters for stacked autoencoder. Where sub-network is the multiple layer networks of RNAs and proteins. And we minimize cross entropy loss function using SGD with momentum 0.9 [56]. For each layer of denoising autoencoder, mean squared error is minimized using Adam [58]. We apply dropout training with dropout probability 0.5 during model training [59]. After completing the training process, we extract the learned high-level features both for before and after fine tuning, then they are fed into random forest, the predictors are called SDA-RF and SDA-FT-RF, respectively.

In this study, we implement stacked autoencoder using keras library https://github.com/fchollet/keras. The value of nb_epoch and batch_size are both 100.

### Stacked ensembling

In general, different classifiers have different performance, ensemble learning makes use of multiple classifiers to approximately obtain the optimum target function. How to integrate the individual outputs when implementing the ensembling mechanism is very crucial. Previous studies include majority voting [23] and averaging individual model results [60].

In stacked ensembling, following the deep learning intuition using multiple layers of neural networks, the combining strategy is that the outputs of the level 0 classifiers will be served as training data for another level 1 classifier [61]. Where level 0 is the first layer, and level 1 is the successive layer. The level 1 classifier will figure out how to combine the results from individual classifiers. In this study, the outputs of the level 0 classifiers is predicted probability score, and level 1 classifier is logistic regression. When weights of logistic regression for all individual classifiers is the same, then it is like averaging strategy. When only one weight is non-zero, it is equivalent to majority voting strategy.

3 $$ P_{\mathbf{w}}(y = {\pm}1|\mathbf{x}) = \frac{1}{1 + \exp(-y\mathbf{w}^{T}\mathbf{x})}  $$

where **x** is vector of output probability for SDA-FT-RF, RPISeq-RF and SDA-RF, and **w** is the weight vector for the three classifiers. In this study, implementation of logistic regression is from Scikit-learn [62].

### Evaluation criteria

In this study, we classify protein and ncRNA pairs to be interacting or not. We follow the widely used evaluation measure by means of the classification accuracy, precision, sensitivity, specificity and the Matthews correlation coefficient (MCC) as defined respectively by:

4 $$\begin{array}{@{}rcl@{}} Accuracy &=& \frac{TP + TN}{TP + TN + FP + FN} \end{array} $$

5 $$\begin{array}{@{}rcl@{}} Sensitivity &=& \frac{TP}{TP + FN} \end{array} $$

6 $$\begin{array}{@{}rcl@{}} Specificity &=& \frac{TN}{TN + FP} \end{array} $$

7 $$\begin{array}{@{}rcl@{}} Precision &=& \frac{TP}{TP + FP} \end{array} $$

8 $${} MCC = \frac{TP \times TN - FP \times FN}{\sqrt{(TP + FP)(TP + FN)(TN + FP)(TN + FN)}}  $$

where TP, TN, FP, and FN represents true positive, true negative, false positive, and false negative, respectively. We also exploit Receiver Operating Characteristic (ROC) curve and calculate the area under the ROC curve (AUC). 5-fold cross-validation is used to evaluate the performance of IPMiner. To guarantee the unbiased comparison, the testing and training datasets do not overlap with each other.

## Abbreviations

RBPs, RNA binding proteins; ncRNAs, non-coding RNAs; lncRNAs, long non-coding RNAs; GO, gene ontology; IPMiner, interaction pattern miner; RF, random forest; SVM, support vector machine; SDA-RF, stacked denoising autoencoder random forest; SDA-RF, stacked denoising autoencoder fine tunning random forest; ROC, receiver operating characteristic; AUC, the area under the ROC curve; MCC, Matthews correlation coefficient; MCL, Markov clustering; SGD, stochastic gradient descent

## Additional files

Additional file 1
**Figure S1.** The distribution change of significant feature Cysteine abundance after discarding positives without completely available features, where Cysteine abundance is indicated significant for RNA-protein interaction in Pancaldi et al. 2011. (EPS 30 kb)

Additional file 2
**Supplementary text and Table.** Supplementary description for random forest, Table S1, S2 and S3. (PDF 99 kb)

Additional file 3
**Figure S2.** IPMiner’s performance on two lncRNA-protein datasets (RPI419 and RPI325) with lower sequence similarity. a) Performance on lncRNA-protein dataset RPI419 with RNA sequence similarity cut-off 80 % and protein sequence similarity cut-off 50 %. b) Performance on lncRNA-protein dataset RPI325 with RNA sequence similarity cut-off 80 % and protein sequence similarity cut-off 30 %. (EPS 68 kb)

Additional file 4
**Figure S3.** Performance comparison between stacked ensembling and average ensembling on dataset RPI369. (EPS 37 kb)
