# Supplementary material for: IPMiner: hidden ncRNA-protein interaction sequential pattern mining with stacked autoencoder for accurate computational prediction
Source: BMC Genomics. 2016 Aug 9;17:582. doi: 10.1186/s12864-016-2931-8 (PMC4979166; doi:10.1186/s12864-016-2931-8)
Supplement: Additional file 2 — Supplementary text and Table. Supplementary description for random forest, Table S1, S2 and S3. (PDF 99 kb) [file 12864_2016_2931_MOESM2_ESM.pdf]

Supplementary Materials

IPMiner: Hidden ncRNA-protein interaction sequential pattern mining with stacked autoencoder for accurate computational prediction

1 Methods

1.1 Random Forest

Random forest (RF) [1] is grown from multiple unpruned decision trees, each tree is trained on separate bootstrap samples of the training data, and a feature subset sampled independently from the original feature space. In our experiment, Scikit-learn [2] is used to construct RF model, we only set parameter number of trees as 50, and other parameters are default values. The features are preprocessed to have unit variance before training RF and stacked autoencoder.

Table S1: The details of 18 complexes in RPI488.

Complex	Detection method
1FFK	X-RAY DIFFRACTION
1JJ2	X-RAY DIFFRACTION
1GIY	X-RAY DIFFRACTION
3HUW	X-RAY DIFFRACTION
3I8I	X-RAY DIFFRACTION
1J5A	X-RAY DIFFRACTION
2ZJP	X-RAY DIFFRACTION
2R8S	X-RAY DIFFRACTION
2RKJ	X-RAY DIFFRACTION
3CW1	X-RAY DIFFRACTION
1P85	ELECTRON MICROSCOPY
2GYA	ELECTRON MICROSCOPY
2FTC	ELECTRON MICROSCOPY
2ZKQ	ELECTRON MICROSCOPY
2ZKR	ELECTRON MICROSCOPY
3BBN	ELECTRON MICROSCOPY
3BBO	ELECTRON MICROSCOPY
3JYV	ELECTRON MICROSCOPY

Table S2: The performance of IPMiner using different number of neurons in network on RPI488.

network architecture	MCC
256-128-64	**0.784**
256-128-32	0.778
256-64-64	0.779
128-128-64	0.783
512-256-64	0.783
512-64-64	0.783
512-256-128-64	0.783

X-X-X represents the architecture of stacked network, where X denotes the number of neurons in each hidden layer.

Table S3: The predicted performance of trained model from RPI488 on NPInter2.0, RPI367 and RPIntDB dataset after removing similar sequences with threshold 80%. There is no sequences with similarity greater than 80% for protein and RNA sequences between NPInter2.0, RPI367, RPIntDB and RPI488 respectively.

Dataset	Organism	Total # of ncRNA-protein	Predicted # of ncRNA-protein
NPInter2.0	Homo sapiens	6,959	6,766 (97.2%)
	Caenorhabditis elegans	36	15 (41.7%)
	Mus musculus	2,152	2,065 (95.9%)
	Drosophila melanogaster	91	61 (67.0%)
	Saccharomyces cerevisiae	910	862 (94.7%)
	Escherichia coli	202	144 (71.3%)
	Total	10,350	9,913 (95.7%)
RPI367	Homo sapiens	124	91 (73.4%)
	Caenorhabditis elegans	2	2 (100.0%)
	Mus musculus	22	16 (72.7%)
	Drosophila melanogaster	26	22 (84.6%)
	Saccharomyces cerevisiae	119	116 (97.5%)
	Escherichia coli	25	19 (76.0%)
	Total	318	266 (83.6%)
RPIntDB	Total	16,912	14,826 (87.6%)

References

[1] Breiman,L. Random forest. *Machine Learning.* 2001;45: 5-32.

[2] Pedregosa,F. *et al.* Scikit-learn: Machine learning in Python. *J Mach Learn*

Res. 2011;12: 2825-2830.
